# Genetics of Movement Disorders: Lessons from Essential Tremor

**DOI:** 10.3390/neurolint17120206

**Published:** 2025-12-17

**Authors:** Daniele Orsucci

**Affiliations:** Unit of Neurology, San Luca Hospital, Via Lippi-Francesconi, 55100 Lucca, Italy; orsuccid@gmail.com or daniele.orsucci@uslnordovest.toscana.it

Movement disorders comprise a heterogeneous group of neurological conditions shaped by a complex interplay of genetic and environmental factors. Advances in molecular genetics have profoundly expanded our understanding of the mechanisms underlying these disorders, uncovering a multifaceted network of monogenic, polygenic, and environmental contributions. This Special Issue aims to present recent breakthroughs in the genetics of movement disorders, with contributions highlighting how genetic discoveries are transforming both diagnostic strategies and therapeutic approaches.

Until recently, most general neurologists, including many specializing in movement disorders, showed limited engagement with neurogenetics. When encountering patients with suspected hereditary conditions or particularly complex clinical presentations, they would typically refer these cases to university or tertiary referral centers. Within these highly specialized facilities, patients often entered a protracted diagnostic process that could last for years, especially when initial genetic testing yielded negative results. In many cases, a definitive diagnosis remained elusive, leading to significant emotional and practical consequences for patients, including psychological stress, financial strain, and difficulties accessing the widely regarded leading experts when needed.

In recent years, next-generation sequencing (NGS) technologies, particularly whole-exome sequencing (WES), have revolutionized the diagnosis of genetic neurological diseases. NGS-based studies are now widely available and enable the simultaneous analysis of thousands of genes within weeks or months, at a fraction of the cost of traditional Sanger sequencing. These advances have greatly enhanced diagnostic efficiency and accessibility. In many instances, patients no longer need to travel to referral centers, as blood samples can be directly submitted to NGS laboratories for analysis [1].

Advances in genetics, particularly the advent of NGS, have revealed the remarkable complexity of genotype–phenotype correlations in movement disorders. Mutations in a single gene can produce multiple, often divergent phenotypes, posing challenges for clinico-genetic correlation. Phenotypic heterogeneity arises from incomplete penetrance, variable expressivity, and pleiotropy, driven by genetic, epigenetic, and environmental factors. In this context, movement disorder specialists remain essential for defining disease phenotypes, performing deep phenotyping, and interpreting variant pathogenicity, which is critical for accurate diagnosis, management, and genetic counseling [2].

Nevertheless, important challenges remain. The scale and complexity of genomic data necessitate sophisticated bioinformatic tools and careful interpretation. Accurate clinical phenotyping remains indispensable for correlating genetic variants with disease manifestations, and in a significant subset of cases, even extensive genomic studies may not yield a definitive diagnosis. Strong collaboration between clinical neurologists and geneticists is therefore essential to address these interpretative challenges and to translate genetic data into meaningful clinical insights.

It is also important to note that current NGS studies are not capable of detecting certain types of genetic rearrangements, such as nucleotide repeat expansions (e.g., in Huntington’s disease, the most common dominant spinocerebellar ataxias, Friedreich’s ataxia, etc.) and deletions (e.g., those frequently observed in *SPG4*-related spastic paraparesis) [3]. In all these cases, clinical judgment remains essential, and targeted genetic testing for these specific conditions should be performed prior to whole-exome analysis if there is a clinical suspicion of one of these disorders.

A clearly hereditary movement disorder is Essential Tremor (ET), so much so that any clinical neurologist recognizes the absence of a family history as a clue to question the diagnosis. Nevertheless, for many years, genetic studies treated ET as a multifactorial condition, focusing on the analysis of polymorphisms rather than searching for mutations segregating with the phenotype in affected families. Only in recent years some NGS studies have enabled the identification of genetic variants associated with ET, or with tremor phenotypes clinically indistinguishable from it [4,5,6,7,8]. These studies have enabled the association of certain genes (i.e., *CACNA1G*, *CAMTA1*, *FUS*, *GPR151*, *NOTCH2NLC*, *SGCE*, *TENM4*) with familial ET, but more importantly, they have shown that most cases of familial ET, inherited in an autosomal dominant manner, may result from mild variants in a variety of genes. When these same genes carry more deleterious variants, they can give rise to more severe neurological syndromes, still following an autosomal dominant pattern. Thus, the ET phenotype may represent the mild or incomplete manifestation of many other dominant neurogenetic disorders. These findings further underscore the genetic heterogeneity of the condition [8].

In this Special Issue (“Genetics of Movement Disorders” https://www.mdpi.com/journal/neurolint/special_issues/SO5907L00Z accessed on 7 December 2025), the genetic foundations of movement disorders will be reviewed and discussed. We hope that the contributions assembled here will serve as a valuable resource for general neurologists and specialists alike, guiding them through the rapidly evolving landscape of neurogenetics in movement disorders.
